# Genomic Analysis of Factors Associated with Low Prevalence of Antibiotic Resistance in Extraintestinal Pathogenic *Escherichia coli* Sequence Type 95 Strains

**DOI:** 10.1128/mSphere.00390-16

**Published:** 2017-04-05

**Authors:** Craig M. Stephens, Sheila Adams-Sapper, Manraj Sekhon, James R. Johnson, Lee W. Riley

**Affiliations:** a Biology Department and Public Health Program, Santa Clara University, Santa Clara, California, USA; b Division of Infectious Diseases and Vaccinology, School of Public Health, University of California, Berkeley, California, USA; c Veterans Affairs Medical Center and University of Minnesota, Minneapolis, Minnesota, USA; Centers for Disease Control and Prevention

**Keywords:** *Escherichia coli*, ExPEC, ST95, antibiotic resistance, genomics

## Abstract

Extraintestinal pathogenic *Escherichia coli* (ExPEC) strains belonging to multilocus sequence type 95 (ST95) are globally distributed and a common cause of infections in humans and domestic fowl. ST95 isolates generally show a lower prevalence of acquired antimicrobial resistance than other pandemic ExPEC lineages. We took a genomic approach to identify factors that may underlie reduced resistance. We fully assembled genomes for four ST95 isolates representing the four major *fimH*-based lineages within ST95 and also analyzed draft-level genomes from another 82 ST95 isolates, largely from the western United States. The fully assembled genomes of antibiotic-resistant isolates carried resistance genes exclusively on large (>90-kb) IncFIB/IncFII plasmids. These replicons were common in the draft genomes as well, particularly in antibiotic-resistant isolates, but we also observed multiple instances of a smaller (8.3-kb) ampicillin resistance plasmid that had been previously identified in *Salmonella enterica*. Among ST95 isolates, pansusceptibility to antibiotics was significantly associated with the *fimH6* lineage and the presence of homologs of the previously identified 114-kb IncFIB/IncFII plasmid pUTI89, both of which were also associated with reduced carriage of other plasmids. Potential mechanistic explanations for lineage- and plasmid-specific effects on the prevalence of antibiotic resistance within the ST95 group are discussed.

**IMPORTANCE** Antibiotic resistance in bacterial pathogens is a major public health concern. This work was motivated by the observation that only a small proportion of ST95 isolates, a major pandemic lineage of extraintestinal pathogenic *E. coli*, have acquired antibiotic resistance, in contrast to many other pandemic lineages. Understanding bacterial genetic factors that may prevent acquisition of resistance could contribute to the development of new biological, medical, or public health strategies to reduce antibiotic-resistant infections.

## Abstract (precis)

Antibiotic resistance in bacterial pathogens is a major public health concern. This work was motivated by the observation that only a small proportion of ST95 isolates, a major pandemic lineage of extraintestinal pathogenic *E. coli*, have acquired antibiotic resistance, in contrast to many other pandemic lineages. Understanding bacterial genetic factors that may prevent acquisition of resistance could contribute to the development of new biological, medical, or public health strategies to reduce antibiotic-resistant infections.

## INTRODUCTION

Genetically diverse strains of the Gram-negative bacterium *Escherichia coli* are capable of causing many human illnesses, including both gastrointestinal and extraintestinal infections. This species is the most common cause of urinary tract infections (UTIs) (1) and a leading cause of bloodstream infections (BSIs) (2). With few exceptions, *E. coli* strains with an enhanced ability to cause extraintestinal infections—referred to collectively as extraintestinal pathogenic *E. coli* (ExPEC)—are genetically distinct from strains that cause gastrointestinal illness (3). Epidemiological observations have shown that ExPEC strains are derived primarily from a small number of phylogenetic lineages, as defined by multilocus sequence typing (MLST), including sequence types 131 (ST131), ST95, ST69, ST73, and ST393. These STs are responsible for nearly half of all *E. coli* UTIs or BSIs in many regions of the world (4). Comparative genomic analysis of ExPEC strains to date has not conclusively identified essential “pandemicity” genes in these lineages (5), but there is considerable interest in understanding virulence-associated genetic features of pandemic ExPEC lineages.

In several recent surveys of ExPEC isolates from the United States, ST95 was the second most common ST, after ST131. These include reports by Adams-Sapper et al. (6), who examined bloodstream isolates from a San Francisco, CA, hospital; Bannerjee et al. (7), who examined isolates from blood, urine, and other extraintestinal sites from Olmsted County, Minnesota; and Salipante et al. (5), who examined urinary and bloodstream isolates from the University of Washington Hospital system (Seattle, WA). ST131 clinical isolates typically are multidrug resistant (MDR), expressing fluoroquinolone resistance due to chromosomal mutations and, in many instances, extended-spectrum β-lactam resistance due to acquired plasmid-borne or chromosomal genes (8, 9). In contrast, ST95 isolates are much less frequently antibiotic resistant than ST131 strains and many other clonal groups (4). Furthermore, Adams-Sapper et al. (6) noted that, at least in San Francisco, the *fimH6* sublineage of the ST95 group was nearly always pansusceptible and yet very common. This implies, first, that antimicrobial resistance may not be a major factor contributing to the pandemicity of ST95 strains, and second, that there may be something distinctive about *fimH6* strains with respect to acquisition or maintenance of antibiotic resistance.

We describe here the use of whole-genome sequence analysis to explore these implications. We sequenced 44 ST95 bloodstream isolates from San Francisco General Hospital (SFGH) that had been recovered between 2007 and 2011 (6, 10). These initial strains encompassed the four major sublineages of ST95, as defined by alleles of the *fimH* marker. We added more geographic diversity with four ST95 isolates from Minnesota (11) and five from other locations in the United States (12). Finally, we included archived sequences of 33 ST95 isolates from the Seattle area (5) in our analysis. Through this approach, we were able to identify the genetic basis for acquired antibiotic resistance genes in a large United States-based sample of ST95 isolates and to identify genomic differences between pansusceptible and resistant isolates that help explain disparities in resistance within the ST95 group.

## RESULTS

### Genome sequencing and isolate characterization.

**FIG 1:**
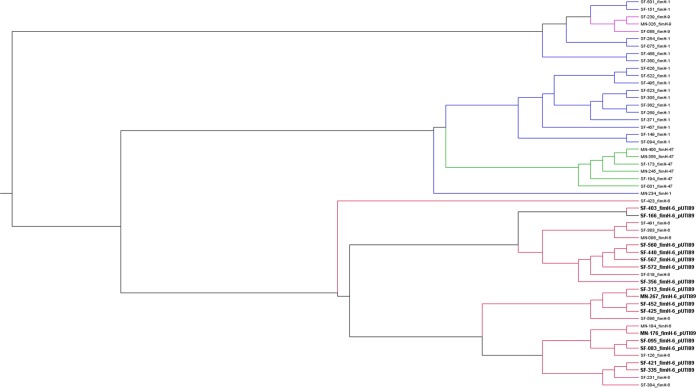
Phylogenetic tree showing relationships between ST95 isolates based on alignment of scaffolded genome assemblies. Raw sequencing reads for each genome were trimmed and filtered for quality control and then assembled to the *E. coli* SF-166 complete genome sequence (GenBank accession no. CPO12633). The resulting genome sequences were aligned using progressiveMauve 2.4.0 (38), and an unrooted tree based on the alignments was generated using Archaeopteryx 0.9920. Isolate names are shown to the right, along with *fimH* type and whether the isolate also contains a pUTI89* plasmid.

A total of 53 *E. coli* ST95 isolates were subjected to whole-genome sequencing on the Illumina MiSeq platform, followed by *de novo* assembly and analysis (Table 1; see Table S1 in the supplemental material). Most (83%) of the isolates were from San Francisco, CA. We also examined archived whole-genome sequences of 33 ST95 isolates from the Seattle, WA, region (5). The *fimH* gene, which encodes an adhesin critical for urinary tract pathogenesis, has proven to be useful as a genetic marker to increase the resolution of MLST with ExPEC strains (13, 14). Examination of *fimH* genotypes from genome sequences allowed assignment of the ST95 isolates to sublineages (Table 1). Alignment of scaffolded assemblies from the draft genome sequences and subsequent tree building (Fig. 1) showed the *fimH* sublineages to be phylogenetically coherent, with the major division being between *fimH1* and *fimH6* isolates, and with the *fimH9* and *fimH47* clusters emerging from separate branches of the *fimH1* group. Among the isolates we sequenced, the *fimH6* genotype was most abundant (48%), followed by the *fimH1* (31%), *fimH47* (17%), and *fimH9* (3%) genotypes.

10.1128/mSphere.00390-16.3 TABLE S1  Overview of *E. coli* ST95 genomes sequenced for this work. Download TABLE S1, DOCX file, 0.1 MB. Copyright © 2017 Stephens et al. 2017 Stephens et al. This content is distributed under the terms of the Creative Commons Attribution 4.0 International license.

**TABLE 1:** Characterization of *E. coli* ST95 isolates and genomes

FimH gene type	Isolate^a^	Antibiotic resistance^b^	No. of plasmid replicons present^c^						No. of small plasmids^d^	Full pUTI89^e^
			IncFI	IncFII	IncB	IncI	IncP	IncQ		
*fimH1*	SF-075	Tet (*tetA*)	2 (B, C)	1				1	3	
	SF-094	Amp (*bla*_TEM-1_)	1 (B)	1					6	
	SF-149	Amp (*bla*_TEM-1_)	1 (B)	1					3	
	SF-151	Amp (*bla*_TEM-1_), Str (*strA*, *strB*), Tet (*tetA*), Sul (*sul2*), Tmp (*dfrA5*)	1 (B)	1				1	2	
	SF-264	Amp (*bla*_TEM-1_), Azm (*mphA*), Chl (*catA1*), Gen (*aac3*), Nor (gyrA*), Str (*aadA1*, *aadA2*, *aadA5*), Tet (*tetA*), Sul (*sul1*), Tmp (*dfrA12*, *dfrA17*)		1			1		3	
	SF-269	Amp (*bla*_TEM-1_)	1 (B)	2	1				5	
	SF-305	Amp (*bla*_TEM-1_)	1 (B)	2					6	
	SF-362	Amp (*bla*_TEM-1_)	1 (B)	1	1				4	
	SF-371	Amp (*bla*_TEM-1_)		1					5	
	SF-380	None	1 (B)	1					2	
	SF-457	Amp (*bla*_TEM-1_)	1 (B)	1					7	
	SF-468	Amp (*bla*_TEM-1_), Cef (*bla*_CTX-M14_), Gen (*aac3*), Str (*strA*, *strB*), Tet (*tetA*), Sul (*sul2*), Tmp (*dfrA17*)	1			1			3	
	SF-495	None	1 (B)	1	1				2	
	SF-501	Amp (*bla*_TEM-1_), Str (*strA*, *strB*), Tet (*tetD*), Sul (*sul2*), Tmp (*dfrA5*)	1 (B)	1				1	2	
	SF-522	Amp (*bla*_TEM-1_), Str (*strA*, *strB*), Tet (*tetB*)		1					6	
	SF-523	None	1 (B)	1					5	
	SF-626	Amp (*bla*_TEM-1_)	1 (B)	2					6	
	MVAST0234	None	1 (B)	1					3	
	UPEC-061	None	1 (B)	1					ND	Yes
	UPEC-094	*bla*_TEM-1_, *dfrA5*		1					ND	
	UPEC-106	None	1 (B)	1					ND	
	UPEC-136	None	1 (B)	1	1				ND	
	UPEC-144	*bla*_TEM-1_, *strA*, *strB*, *sul2*, *dfrA5*	1 (B)	1				1	ND	
	UPEC-185	*bla*_TEM-1_, *strA*, *strB*, *sul2*, *dfrA*^f^	1 (B)	1				1	ND	
	UPEC-249	None	1 (B)	1					ND	
	UPEC-250	*bla*_TEM-1_, *strA*, *strB*, *sul2*, *dfrA5*	1 (B)	1				1	ND	
	UPEC-276	None	1 (B)	1					ND	
*fimH6*	SF-083	None	1 (B)	2					0	Yes
	SF-095	None	1 (B)	1					1	Yes
	SF-126	Amp (*bla*_TEM-1_), Tet (*tetA*)	3 (A, B, C)	1					0	
	SF-166	None	1 (B)	1					0	Yes
	SF-231	Amp (*bla*_TEM-1_), Str (*strA*, *strB*, *aadA5*), Tet (*tetB*), Sul (*sul1*, *sul2*), Tmp (*dfrA17*)	1 (B)	1				1	0	
	SF-313	None	1 (B)	1					0	Yes
	SF-335	Amp (*bla*_TEM-1_)	1 (B)	1	1				2	Yes
	SF-356	None	1 (B)	1					0	Yes
	SF-383	None	1 (B)	1					2	
	SF-384	None	1 (B)	1					0	
	SF-403	None	1 (B)	1					0	Yes
	SF-421	None	1 (B)	1					3	Yes
	SF-423	None	1 (B)	1					0	
	SF-425	None	1 (B)	1					0	Yes
	SF-440	None	1 (B)	1					0	Yes
	SF-452	None	1 (B)	2					0	Yes
	SF-491	None	1 (B)	1					0	
	SF-518	None							0	
	SF-560	None	1 (B)	1					0	Yes
	SF-567	None	1 (B)	1					0	Yes
	SF-572	None	1 (B)	1					0	Yes
	SF-596	Amp (*bla*_TEM-1_), Str (*strA*, *strB*, *aadA5*), Tet (*tetA*), Sul (*sul1*, *sul2*), Tmp (*dfrA17*)	2 (A, B)	2					2	
	MVAST0098	None	1 (B)	1					0	
	MVAST0176	Amp (*bla*_TEM-1_)	1 (B)	2					0	Yes
	USVAST184	None	1 (B)	1	1				0	
	USVAST267	None	1 (B)	1	1	1			2	Yes
	Blood-11-0031	None		1					ND	
	UPEC-007	*bla*_TEM-1_, *strB*	3 (A, B, C)		1				ND	
	UPEC-008	None							ND	
	UPEC-048	*bla*_TEM-1_	1 (B)	1	1				ND	
	UPEC-051	None	1 (B)	1					ND	
	UPEC-072	None							ND	
	UPEC-073	None	1 (B)	1					ND	
	UPEC-075	None	1 (B)	1					ND	Yes
	UPEC-098	None^f^	1 (B)	1					ND	Yes
	UPEC-124	None^f^							ND	
	UPEC-131	None^f^	1 (B)	1					ND	Yes
	UPEC-139	None	1 (B)	1					ND	Yes
	UPEC-157	None^f^	1 (B)						ND	
	UPEC-197	*bla*_TEM-1_							ND	
	UPEC-255	None	1 (B)	1					ND	Yes
*fimH9*	SF-088	Amp (*bla*_TEM-1_), Str (*strA*, *strB*), Sul (*sul2*), Tmp (*dfrA5*)	1 (B)	1				1	2	
	SF-239	None	1 (B)	1					0	
	MVAST326	None	1 (B)	1		1			4	
*fimH47*	SF-001	Amp (*bla*_TEM-1_)	1 (B)	1	1				0	
	SF-173	Amp (*bla*_TEM-1_), Azm (*mphA*), Str (*aadA2*), Sul (*sul1*), Tmp (*dfrA12*)		1					0	
	SF-194	Amp (*bla*_TEM-1_), Chl (*catA1*), Str (*aadA1*), Sul (*sul1*), Tet (*tetA*)	3 (A, B, C)	1			1		0	
	USVAST245	None	1 (B)	1					2	
	USVAST356	Amp (*bla*_TEM-1_)	2 (A, B)	1					0	
	USVAST406	Amp (*bla*_TEM-1_)	3 (A, B, C)	1					0	
	Blood-08-0493	None	1 (B)	1					ND	Yes
	Blood −08 to 654	None	1 (B)	1					ND	Yes
	Blood-09-0751	*bla*_TEM-1_, *strA*, *strB*, *sul2*							ND	
	Blood-10-687	None	1 (B)	1					ND	Yes
	UPEC-076	None							ND	
	UPEC-120	None	1 (B)	1					ND	
	UPEC-129	None	1 (B)	1					ND	Yes
	UPEC-169	None	1 (B)	1					ND	Yes
	UPEC-209	*bla*_TEM-1_, *strA*, *strB*, *sul2*, *dfrA14*, *tetB*	1 (B)	1	1				ND	

a SF isolates were from San Francisco General Hospital (6), MVAST isolates were from Minneapolis (11), USVAST isolates were from other states in the United States (10), and “Blood” and “UPEC” isolates were from Seattle (5).

b Antibiotic resistance phenotypes are shown for all strains other than those from Seattle, with the ResFinder-identified gene(s) (20) presumed responsible shown in parentheses. For Seattle isolates, only the genes identified are shown, as phenotypes were not independently confirmed in this study. Antibiotic abbreviations: Amp, ampicillin; Azm, azithromycin; Cef, cephalothin; Chl, chloramphenicol; Gen, gentamicin; Nor, norfloxacin; Str, streptomycin; Tet, tetracycline; Sul, sulfamethoxazole; Tmp, trimethoprim.

c Plasmid replicons were predicted with PlasmidFinder (25).

d The number of small plasmids was determined from sequence assemblies, as described in Materials and Methods. Seattle isolates are listed as “ND” (no data), as these genomes were downloaded as assembled contigs without topology or coverage data, which precluded identification of small plasmids.

e Prediction of the presence of pUTI89 is described in Materials and Methods.

f The presence of resistance genes is discordant with phenotypes reported in reference 5: UPEC-007 (reported as Tet^r^), UPEC-098 (reported as Amp^r^ Sul^r^ Tmp^r^), UPEC-124 (reported as Amp^r^ Sul^r^ Tmp^r^), UPEC-131 (reported as Amp^r^ Cef^r^ Tet^r^), UPEC-144 (not reported as Sul^r^ Tmp^r^), UPEC-157 (reported as Amp^r^), UPEC-185 (not reported as Amp^r^ Sul^r^ Tmp^r^).

Based on their *fimH* genotype and antibiotic resistance phenotypes, four ST95 isolates from San Francisco were selected for long-read, single-molecule real-time (SMRT) sequencing (Pacific Biosciences) to develop high-quality, fully assembled reference genomes (15). The complete genomes were from SF-166 (*fimH6* [pansusceptible, as defined in Materials and Methods]), SF-173 (*fimH47* [MDR]), SF-468 (*fimH1* [MDR]), and SF-088 (*fimH9* [MDR]). Key characteristics of the genome sequences are presented in Table S1. The complete chromosomes of the four strains aligned readily, with >99.9% identity across most of the genome (see Fig. S1 in the supplemental material). Most breaks in the alignment between the complete chromosomes involved prophages. Other discontinuities in the alignments involved loci for synthesis of O-antigens and P-pili. O-antigen loci are common sites of recombination and variation in *E. coli*; the ST95 isolates examined in this work varied in the O-antigen loci and predicted serotypes, even within *fimH* types (data not shown). Additionally, a 40-kb segment located at approximately 0.8 Mb in SF-166 and SF-088 was moved to 4.6 Mb in SF-468 and SF-173. This segment includes the *pap* genes encoding P-pili, an important ExPEC virulence factor (16).

10.1128/mSphere.00390-16.1 FIG S1  Alignment of finished chromosomes from SMRT-sequenced *E. coli* ST95 isolates. Sequence alignment was done with the progressiveMAUVE (38) plug-in for Geneious; the circular map was generated with BRIG (39). Approximate numbering is shown inside the map. From inside out, black indicates GC content, red SF-166 (*fimH6*), violet SF-088 (*fimH9*), green SF-173 (*fimH47*), and blue SF-468 (*fimH1*). Labeled are *oriC* (black), the loci for O-antigen synthesis (orange) and P-pilus synthesis (*pap* genes [green]), and various prophages, including the two highly conserved phages SFII and HK629. Download FIG S1, DOCX file, 0.3 MB. Copyright © 2017 Stephens et al. 2017 Stephens et al. This content is distributed under the terms of the Creative Commons Attribution 4.0 International license.

Because clustered regularly interspaced short palindromic repeat (CRISPR) elements are thought to impact mobile gene acquisition (including plasmids bearing antibiotic resistance) in bacterial genomes (17), we examined CRISPR loci in the fully assembled ST95 genomes. All four isolates lacked the CRISPR1 locus and had only two imperfect and identical spacers at CRISPR2 (located at ~1.12 Mb), as has been observed previously in ST95 isolates (18). CRISPR3 and CRISPR4 (located at ~3.2 Mb) were present, with a variable number of repeats (for CRISPR3, SF-088, 4, SF-166, 7, SF-173, 8, and SF-468, 5; for CRISPR4, SF-088, 2, SF-166, 6, SF-173, 6, and SF-468, 6). When draft genomes were examined, there was some variation in repeat numbers within *fimH* lineages as well (data not shown).

SMRT sequencing allowed identification of DNA methylation patterns (19). All four genomes displayed adenine methylation (m6A) at GATC sites, attributable to Dam DNA methyltransferase (see Table S2 in the supplemental material). The SF-468 and SF-173 isolates shared an additional m6A motif, as did the SF-166 and SF-088 isolates. SF-088 showed m6A methylation at two additional sites. All motifs showing m6A methylation were nearly 100% methylated. The SF-166 genome was the only one to show cytosine (m4C) methylation, at roughly one out of eight RCCGGY sequences. The SF-166 genome contains three annotated DNA-cytosine DNA-methyltransferase genes: a homolog of *dcm* (located at ~2.1 Mb) that was present in all of the other ST95 genomes sequenced, a component of a restriction-modification system (located at 4.12 Mb) found in all *fimH6* and *fimH9* isolates, and a 1.2-kb gene located at 4.16 Mb in SF-166 that was universal in *fimH6* isolates but absent in the non-*fimH6* ST95 genomes examined. The latter gene is hypothesized to be responsible for the unique m4C methylation observed in SF-166, but this was not confirmed experimentally.

10.1128/mSphere.00390-16.4 TABLE S2  DNA methylation motifs identified in ST95 isolates through SMRT sequencing. Download TABLE S2, DOCX file, 0.1 MB. Copyright © 2017 Stephens et al. 2017 Stephens et al. This content is distributed under the terms of the Creative Commons Attribution 4.0 International license.

### Antibiotic resistance.

Thirty-four (40%) of the 86 genomes we examined contained at least one resistance gene, as identified by ResFinder (20). Resistance genes were detected for seven classes of antimicrobial agents (Table 2). For the 53 isolates we sequenced and assessed for resistance, predictions by ResFinder correlated well with observed phenotypes. For the Seattle isolates, intact resistance genes were assumed to confer the expected phenotype. Resistance genes were not evenly distributed among *fimH* sublineages: only 8 (20%) of 41 *fimH6* isolates contained one or more acquired resistance genes, versus 1 (33%) of 3 *fimH9* isolates, 7 (47%) of 15 *fimH47* isolates, and 18 (67%) of 27 *fimH1* isolates (for *fimH6* versus non-*fimH6* isolates, *P* < 0.001). With respect to geographic origin, the Seattle ST95 isolates were significantly less likely to contain genes for β-lactam and tetracycline resistance (*P* = 0.01 and 0.02, respectively) than the San Francisco isolates, a result attributable to the non-*fimH6* component of the population.

**TABLE 2:** Summary of antibiotic resistance gene frequencies in ST95 genomes

Antibiotic class	Resistance gene(s) identified	Proportion (%) of genes in:								
		All isolates			*fimH6* isolates			Non-*fimH6* isolates		
		San Francisco	Seattle	Other	San Francisco	Seattle	Other	San Francisco	Seattle	Other
β-Lactams	*bla*_TEM-1_, *bla*_CTX-M14_	21/44 (48)	9/33 (27)	3/9	4/22 (18)	3/15 (20)	1/4	17/22 (77)	6/18 (33)	2/5
Aminoglycosides	*aadA1*,* aadA2*, *aadA5*, *aac3*, *strA*, *strB*	10/44 (23)	6/33 (18)	0	2/22 (9)	1/15 (7)	0	8/22 (36)	5/18 (28)	0
Tetracyclines	*tetA*, *tetB*, *tetD*	9/44 (20)	1/33 (3)	0	3/22 (14)	0/15	0	6/22 (27)	1/18 (6)	0
Sulfonamides	*sul1*, *sul2*	9/44 (20)	5/33 (15)	0	2/22 (9)	0/15	0	7/22 (32)	5/18 (28)	0
Trimethoprim	*dfrA5*, *dfrA12*, *dfrA17*	8/44 (18)	5/33 (15)	0	2/22 (9)	0/15	0	6/22 (27)	5/18 (28)	0
Macrolides	*mphA*	2/44 (5)	0/33	0	0/22	0/15	0	2/22 (9)	0/18	0
Chloramphenicols	*catA1*	2/44 (5)	0/33	0	0/22	0/15	0	2/22 (9)	0/18	0

The aminopenicillin resistance gene *bla*_TEM-1_, found in 38% of isolates, was the most common acquired resistance gene. Most *bla*_TEM-1_ genes were located in the context of Tn*3* mobile elements. The only extended-spectrum β-lactamase (ESBL) gene identified, *bla*_CTX-M-14_, was on plasmid pSF-468-2 in strain SF-468, which also contained *bla*_TEM-1_ on a separate plasmid (pSF-468-1). Among the 33 ampicillin-resistant isolates, 15 were resistant to at least three classes of antibiotics. The MDR isolates accounted for nearly all detected genes conferring resistance to aminoglycosides, tetracyclines, and sulfamethoxazole/trimethoprim (Table 1). Sulfonamide and trimethoprim resistance genes were typically coresident (occurring together in 12 of 15 isolates with either type of gene) and in close proximity on the same contig. One strain (SF-264) was also resistant to fluoroquinolones due to the presence of typical chromosomal mutations (in *gyrA*, Ser83-to-Leu and Asp87-to-Asn; in *parC*, Ser80-to-Ile) encountered in fluoroquinolone-resistant clinical *E. coli* isolates (21).

### Plasmid content of ST95 genomes.

Each of the SMRT-sequenced and fully assembled ST95 genomes contained at least one circular plasmid larger than 90 kb. These large plasmids all contained one or more known Inc replicons (Table S1), and with one exception (pSF-166-1), all of the large plasmids contained antibiotic resistance genes. Furthermore, all of the resistance genes in the three sequenced MDR isolates were contained on large plasmids. pSF-468-1 carried *bla*_TEM-1_ in a Tn*3*-type transposon adjacent to a 17-kb region containing genes for resistance to streptomycin (*strAB*), sulfonamides (*sul2*), trimethoprim (*dfrA17*), and tetracycline (*tetA*). pSF-088-1 carried *bla*_TEM-1_, *sul2*, *dfrA5*, *strA*, and *strB* in an 11-kb region, while pSF-173-1 included *dfrA12*, *aadA2*, *sul1*, and *mphA* in a 10-kb region. The large IncF plasmids (pSF-468-1, pSF-088-1, pSF-173-1, and pSF-166-1) shared roughly 40 kb of sequence encoding conserved replication and conjugation functions. pSF-468-1 also shared with pSF-088-1 another ~45 kb of sequence that included genes encoding potential virulence-associated factors, such the Sit manganese-iron transport system and genes for aerobactin siderophore production and uptake. Virulence factors in the ST95 genomes will be reported in greater detail elsewhere.

pSF-166-1, the 114-kb plasmid from pansusceptible strain SF-166, was the only large plasmid not encoding antibiotic resistance and the only one with a full-length match (>98% identity, 100% coverage) to previously described plasmids: pUTI89 from uropathogenic *E. coli* strain UTI89 (22), plasmid pRS218 from meningitis-associated *E. coli* strain RS218 (23, 24), and plasmid pEC14_114 from uropathogenic strain EC14 (25). This plasmid carries several candidate virulence factors and has been associated with virulence in a mouse UTI model (26) and a rat model for neonatal meningitis (24). For practical purposes, we will refer to this group of plasmids and the close relatives identified in this work as pUTI89* unless greater specificity is needed.

Examination of our draft genomes showed that pUTI89* was common. When pUTI89 was used as the scaffold for reference-guided assemblies of reads from all 53 isolates we sequenced, a complete version of pUTI89* (defined as >98% of pUTI89 assembled) was likely present in 16 isolates—14 from San Francisco, one from Sacramento, and one from Minnesota (Table 1)—all of which were *fimH6* strains. Among the Seattle ST95 isolates (see Materials and Methods), 11/33 likely contained pUTI89*, including 1/9 *fimH1* isolates, 5/15 *fimH6* isolates, and 5/9 *fimH47* isolates. Overall, pUTI89* was likely present in 27 (32%) of 86 ST95 genomes we examined. It was most common in *fimH6* isolates (21/41 [51%]), less common in *fimH47* isolates (5/15 [33%]), and rare in *fimH1* isolates (1/29 [3%]) (for the three-group comparison, by χ^2^ test, *P* = 0.0014).

pUTI89*-containing isolates rarely contained acquired antibiotic resistance genes (2/27 [7%]) compared to the ST95 isolates lacking pUTI89* (27/59 [46%]) (*P* < 0.001). Because most pUTI89*-containing isolates were also in the *fimH6* sublineage, and *fimH6* isolates were also less likely to be resistant than non-*fimH6* isolates, we examined whether these traits were independently associated with resistance frequency in this population of isolates (Table 3). Among *fimH6* isolates, resistance genes were less common among isolates with pUTI89* than among those without pUTI89* (2/21 [10%] versus 6/20 [30%]), but the difference was not significant (*P* = 0.10). Among non-*fimH6* isolates, pUTI89* was also associated with reduced resistance frequency (0/6 resistant isolates containing pUTI89 versus 26/39 resistant isolates lacking pUTI89; *P* = 0.002). Comparing all isolates lacking pUTI89*, *fimH6* isolates were still less likely to be resistant than non-*fimH6* isolates (6/20 [70%] versus 26/39 [33%]; *P* = 0.007). These data suggest that both the *fimH* genotype (specifically *fimH6*) and the presence of pUTI89* may be associated with reduced carriage of antibiotic resistance genes.

**TABLE 3:** Relationship between *fimH* type, pUTI89*, and antibiotic resistance

Isolates	No. of isolates	Proportion (%) antibiotic resistant with:		*P* value^a^
		pUTI89* present	pUTI89* absent	
Total	86	2/27 (7)	32/59 (54)	<0.001
*fimH*6 isolates	41	2/21 (9.5)	6/20 (30)^b^	0.13
Non-*fimH6* isolates	45	0/6 (0)	26/39 (67)^b^	0.003

a *P* values were calculated using the chi-square test.

b For prevalence of resistance among *fimH*6 versus non-*fimH*6 isolates without pUTI89*, *P* = 0.01.

The published sequences of pUTI89 and pRS218 differ by only 16 single nucleotide polymorphisms (SNPs). Alignment of the assembled sequences from this study showed that pSF-166-1 differs from pUTI89 by 32 SNPs and from pRS218 by 40 SNPs. Many of the plasmids from San Francisco isolates were more closely related, including pSF-403-1, pSF-420-1, and pSF-440-1 (7 SNPs), which were obtained from separate patients in late 2009 and early 2010, and pSF-560-1 and pSF-567-1 (6 SNPs), obtained from separate patients in October 2010. Given the expected rate of false-positive SNPs in assemblies from MiSeq data (1 × 10^−4^) (27) at comparable coverage, the plasmids in these isolates may in fact be identical.

Although large plasmids were assembled readily from SMRT sequencing data, they rarely assembled as circular contigs from shorter MiSeq reads, so it was not possible to reliably assess the presence of such plasmids in draft genomes. As an alternative, we used PlasmidFinder (28) to identify replicons from various incompatibility (Inc) groups in draft genome sequences. A total of 190 IncF (IA, IB, IC, or II), IncB, IncI, IncP, or IncQ plasmid replicons were detected in the 86 ST95 genomes examined (mean, 2.2 per genome; standard error of the mean [SEM], 0.11; range, 0 to 5) (Table 1). PlasmidFinder failed to identify plasmid replicons in only five genomes (6%), four of which were *fimH6* strains. Most isolates (83%) contained both IncFIB and IncFII replicons (Table 1); only 9/86 lacked an IncFII replicon, and only 12/86 lacked an IncFIB replicon. The mean number of Inc replicons per strain did not differ significantly by *fimH* lineage. However, replicon type exhibited a borderline significant association with *fimH* lineage: *fimH1* isolates more frequently contained replicons other than FIB and FII (13/27 [48%]) than did *fimH6* (8/41 [20%]) or *fimH47* (5/15 [33%]) isolates (*P* = 0.051).

Because in the draft genomes most large plasmids did not assemble as single contigs, whether antibiotic resistance genes were plasmid associated could not always be determined definitively. However, 35 (64%) of 55 identified antibiotic resistance genes were located on the same contigs as Inc replicons or on pAnkS (see below), suggesting that most resistance genes were plasmid-borne, similar to the pattern observed in the fully assembled genomes.

Strains containing pUTI89* had the same mean number of Inc replicons (2.2/strain) as those lacking pUTI89* (Table 4). However, for pUTI89*-containing isolates, nearly all Inc replicons were IncFIB or IncFII and were presumably present on pUTI89 itself. Isolates without a complete pUTI89 were more likely to contain non-IncFIB/FII replicons or small plasmids (37/59 [63%]) than were pUTI89*-containing isolates (4/27 [15%]) (*P* < 0.001) (Table 4). Thus, the presence of pUTI89* was associated with a significantly lower prevalence of other plasmids.

**TABLE 4:** Plasmid content in isolates containing or lacking the pUTI89-like plasmid

Genome group	Plasmid feature(s) analyzed	Isolates with pUTI89*		Isolates with partial or no pUTI89-like plasmid		*P* value
		No. of isolates	Mean (SEM)	No. of isolates	Mean (SEM)	
ST95 isolates sequenced in this work	Total Inc replicons	16	2.4 (0.2)	37	2.5 (0.2)	0.54
	Inc replicons, excluding IncFIB/IncFII	16	0.2 (0.1)	37	0.7 (0.1)	0.03
	Small plasmids	16	0.5 (0.2)	37	2.2 (0.4)	0.006
All ST95 genomes examined	Total Inc replicons	26	2.2 (0.1)	60	2.2 (0.2)	0.99
	Inc replicons excluding IncFIB/IncFII	26	0.1 (0.1)	60	0.6 (0.1)	0.004

Genomes of pansusceptible ST95 isolates were less likely to contain plasmids than those of resistant strains: the mean number of Inc replicons was lower for isolates lacking resistance genes (mean, 1.9) than for those containing one or more resistance genes (mean, 2.7) (*P* < 0.001 by Student’s *t* test). In the genomes we sequenced, the number of small plasmids was also lower in isolates lacking antibiotic resistance (mean of 0.9 small plasmids/isolate) (*P* = 0.002). Included among the 52 pansusceptible strains were five that lacked Inc replicons and small plasmids altogether.

### Small plasmids in ST95 genomes.

Of the 53 ST95 genomes we sequenced, 26 (49%) contained small plasmids (Table 1), appearing in the draft genome assemblies as circular contigs of less than 10 kb (see Materials and Methods). PlasmidFinder identified some of these as containing replicons of colicin-producing plasmids, but most were not flagged by PlasmidFinder and lacked the Inc replicons enumerated above. Verification that these contigs represented small plasmids was demonstrated by isolation of plasmid DNA in the laboratory (see Fig. S2 in the supplemental material). *fimH1* isolates were much more likely to contain at least one small plasmid (17/18 [94%]) than *fimH6* isolates (6/26 [23%]) (chi-square test, *P* < 0.001). *fimH1* isolates also contained more small plasmids per isolate (3.8/isolate; SEM, 0.4; range, 2 to 7) than *fimH6* isolates (mean, 0.5; SEM, 0.2; range, 0 to 3). Isolates containing pUTI89* (many of which are *fimH6* strains) also contained significantly fewer small plasmids than isolates lacking pUTI89* (Table 4).

10.1128/mSphere.00390-16.2 FIG S2  Visualization of small plasmids from ST95 isolates. Lanes: 1, SF-403, no small plasmids predicted; 2, SF-95, plasmid predicted at 5.7 kb; 3, SF-335, plasmids predicted at 5.8 and 5.3 kb; 4 SF-383, plasmids predicted at 5.7 and 5.2 kb; 5, SF-149, plasmids predicted at 8.4 (pAnkS, indicated by *), 4.1, and 1.6 kb; 6, recombinant *E. coli* TOP10 strain with Amp^r^ plasmid from SF-149 (pAnkS); 7, SF-94, plasmids predicted at 10.3, 8.4 (pAnkS), 4.1, and 1.6 kb; 8, recombinant *E. coli* TOP10 strain with Amp^r^ plasmid from SF-94 (pAnkS); 9, SF-522, plasmids predicted at 10.4, 8.4 (pAnkS), 5.7, 5.2, 4.1, and 1.6 kb; 10, recombinant *E. coli* TOP10 with Amp^r^ plasmid from SF-522 (pAnkS). Note that the 1-kb ladder (New England Biolabs) on either side is linear DNA and migrates more slowly than circular supercoiled plasmids. Download FIG S2, DOCX file, 0.2 MB. Copyright © 2017 Stephens et al. 2017 Stephens et al. This content is distributed under the terms of the Creative Commons Attribution 4.0 International license.

The vast majority of small plasmids identified had no antibiotic resistance genes or known virulence factor genes. An exception was an 8.3-kb plasmid encoding ampicillin resistance that was present in six *fimH1* isolates (SF-94, -149, -305, -457, -522, and -626), isolated over a 4-year period (2007 to 2011). This plasmid and the accompanying β-lactam resistance could be moved into laboratory *E. coli* strains by transformation, allowing it to be separated from other small plasmids present in the clinical isolates (Fig. S2). All ST95 isolates containing the 8.3-kb plasmid showed an elevated cephalothin MIC (32 μg/ml versus 12 μg/ml for other strains containing *bla*_TEM-1_), perhaps due to an increased *bla*_TEM-1_ gene dosage relative to larger, lower-copy-number plasmids in other ST95 strains.

## DISCUSSION

The work presented here explores genomic diversity and antibiotic resistance in the *E. coli* ST95 lineage. Our results suggest that most antibiotic resistance in ST95 strains is associated with large conjugal plasmids. In the SMRT-sequenced SF-088, SF-173, and SF-468 strains, all resistance genes were borne on large multireplicon plasmids. This likely was true also for isolates sequenced to the draft level, since in them most resistance genes were found on contigs containing IncF replicons, as was the case for resistance plasmids in the SMRT-sequenced strains. Intriguing exceptions included six San Francisco isolates containing an 8.3-kb plasmid nearly identical to pAnkS, previously reported in *Salmonella enterica* isolates in Turkey (29) and Uruguay (30). None of the non-San Francisco isolates examined contained a pAnkS-like plasmid, but the temporal and geographic distribution of isolates was far from comprehensive. The pAnkS-containing strains were all isolated between 2007 and 2011; determination of whether this plasmid is still contributing to aminopenicillin resistance in San Francisco or elsewhere warrants further effort.

We also sought to shed light on why ST95 strains are less frequently antibiotic resistant than many other major ExPEC lineages. In previous work, among ST95 clinical isolates from San Francisco the *fimH6* sublineage was most closely associated with pansusceptibility (6). The present analysis of an expanded set of isolates confirmed that *fimH6* isolates were significantly less likely than other ST95 isolates to contain antibiotic resistance genes. Although antibiotic resistance in ST95 isolates derived largely from acquired, plasmid-borne genes, it is conceivable that both chromosomal and plasmid composition could impact the likelihood of acquiring resistance. Indeed, analysis of data in Table 3 suggests that both the *fimH6* genotype and pUTI89* are associated with a reduced likelihood of resistance.

Chromosomal elements in *fimH6* strains such as CRISPR or restriction-modification systems might restrict entry of new plasmids. The ST95 genomes examined here lacked CRISPR1 and contained only a rudimentary CRISPR2, as is common in the *E. coli* B2 group that includes ST95 (18). The CRISPR3 and CRISPR4 loci varied somewhat within and between the *fimH* lineages within ST95. No correlation was observed between the number of spacers or the sequences of spacers in ST95 isolates and their resistance to antibiotics or the presence of particular plasmids. Thus, there is currently no evidence to suggest that CRISPR-mediated effects on DNA acquisition could account for the patterns of antibiotic resistance observed in this work.

Multiple restriction-modification systems are found in each of the completely sequenced ST95 isolates according to REBASE (31). The genome of pansusceptible isolate SF-166 contained two *fimH6*-specific chromosomal regions: an ~12-kb segment at 4.11 Mb that includes a homolog of the Eco31 restriction-modification system (including separate adenine- and cytosine-specific DNA methyltransferases) and a 31-kb segment at 4.15 Mb that includes a DNA-cytosine methyltransferase. The pUTI89* plasmid in SF-166 is also annotated to encode a putative DNA methyltransferase of unknown specificity. Analysis of genome methylation patterns showed no adenine methylation unique to SF-166, but this was the only isolate exhibiting cytosine methylation, and a chromosomally encoded putative cytosine DNA-methyltransferase unique to the *fimH6* lineage was identified. Whether cytosine methylation in *fimH6* strains has any role in limiting plasmid entry, and thereby affecting acquisition of antibiotic resistance, is an open question warranting further study.

Many ST95 isolates, particularly within the *fimH6* sublineage, contained a large multireplicon plasmid nearly identical to pUTI89, designated here generically as pUTI89*. Our data showed that the presence of pUTI89* was correlated with a lower likelihood of antibiotic resistance (Table 3) and a reduced content of other plasmids (Table 4). The preferential association of pUTI89* with the *fimH6* sublineage seems likely to at least partially explain why pansusceptibility is so common in this group (6).

We hypothesize that the pUTI89* plasmid (IncF) inhibits the acquisition or maintenance of other plasmids in the same cell. Incompatibility due to replicon competition could reduce the pool of resistance-bearing plasmids able to establish themselves in cells already containing the multireplicon pUTI89*. For example, in the ST95 strains we examined, *bla*_TEM-1_ was usually located on contigs containing IncF replicons, and such plasmids would be incompatible with a resident pUTI89*. Resistance genes also could be introduced on plasmids from incompatibility groups other than IncF: e.g., the *bla*_TEM-1_-carrying IncB plasmid in pUTI89*-containing isolate SF-335. However, strains containing pUTI89* also were also less likely to harbor plasmids from non-IncF incompatibility groups (Table 4). Thus, factors other than replicon competition may be operative and should be tested experimentally.

Several pUTI89-like plasmids were known previously from the literature. Plasmid pRS218 is derived from *E. coli* strain RS218, which was originally isolated from a neonatal meningitis case in San Francisco in 1974 (32), roughly three decades earlier and thousands of miles separated from the UPEC strains bearing the closely related pUTI89 and pEC14_114 plasmids, which were isolated in St. Louis, MO, and St. Paul, MN, respectively. Both DebRoy et al. (25) and Cusumano et al. (26) have presented PCR-based evidence that pUTI89-like plasmids are common in UTI isolates. pUTI89 and the closely related pRS218 have also been shown to have roles in pathogenesis. A UTI89 derivative cured of pUTI89 was impaired in a mouse UTI virulence model (26), and an RS218 derivative cured of pRS218 was impaired in a rat pup model of neonatal meningitis (23). Genes on pUTI89* that could be involved in pathogenesis include those encoding a potential cytotoxin (*senB*) and systems possibly involved in iron uptake (*cjr*) (26). More detailed dissection of this plasmid has not yet been done due to difficulties working with it. pUTI89 was found to be highly stable during laboratory culture, and presumably is “in the wild” as well, due at least in part to a stabilization system related to the ParM ATPase (33) that prevents plasmid loss (26).

In summary, genomic analysis of ST95 ExPEC strains revealed an extensive plasmidome accounting for acquired antibiotic resistance and an intriguing phenomenon in which a large plasmid (pUTI89), perhaps in collaboration with chromosomal genes, may inhibit a subset of ST95 strains from acquiring other plasmids and associated antibiotic resistance. A deeper understanding of these pansusceptible strains could potentially be exploited to devise biological strategies to combat drug-resistant Gram-negative bacterial infections.

## MATERIALS AND METHODS

### Strains and media.

The 44 *E. coli* ST95 isolates with “SF” designations (Table 1) were drawn from the collection of Gram-negative bacterial strains isolated from bloodstream infections at San Francisco General Hospital (SFGH) between 2007 and 2011, as described by Adams-Sapper et al. (10). Isolates MVAST0098 (urine), MVAST0176 (blood), MVAST0234 (urine), and MVAST0326 (urine) were obtained at the Minneapolis Veterans Affairs Hospital in Minneapolis, MN, in 2010 and 2011 (11). Isolates designated “USVAST” were obtained in 2011 in VA hospitals from the following locations as described by Colpan et al. (12): USVAST184, Ann Arbor, MI; USVAST245, Seattle, WA; USVAST267, Sacramento, CA; USVAST356, Dallas, TX; and USVAST406, Jackson, MS. All of the USVAST isolates were obtained from urine specimens. Strains were routinely cultivated on Luria-Bertani agar. Antimicrobial susceptibility testing of the SFGH isolates (10) was performed by a Microscan WalkAway Gram-negative panel (Dade Behring, Inc., Siemens USA, Deerfield, IL). Extended-spectrum β-lactamase (ESBL) production was confirmed by a double-disc diffusion assay according to 2011 Clinical and Laboratory Standards Institute guidelines, based on cefotaxime and ceftazidime in Luria-Bertani agar plates with and without clavulanic acid. “Pansusceptible” was defined herein as susceptibility to β-lactams (ampicillin and cephalothin), aminoglycosides (gentamicin, kanamycin, and streptomycin), chloramphenicol, quinolones (nalidixic acid and norfloxacin), macrolides (azithromycin), tetracyclines, sulfonamides (sulfamethoxazole), and trimethoprim. “Multidrug resistant” (MDR) was defined herein as resistance to at least three of these classes.

### Genomic DNA sequencing.

Genomic DNA was prepared from all strains using the Qiagen blood and tissue DNeasy kit. Library preparation and sequencing using single-molecule real-time (SMRT) sequencing technology (Pacific Biosciences) have been described elsewhere (15). Analysis of DNA methylation patterns in the SMRT data (19) was done using analysis tools provided by the manufacturer. Library preparation for the MiSeq platform followed a standard protocol for Illumina-compatible libraries (Wafergen Biosystems). Samples were fragmented by a Covaris S220 ultrasonicator to generate an average insert size of 800 bp. After appropriate fragmentation was verified by an Agilent Bioanalyzer, samples were loaded on the Wafergen Apollo 324 NGS Library Prep system. Wafergen PrepX library kits were used for end repair, A-tail addition, adapter ligation, and size selection using AMPure XP beads. Sample concentration was quantified with a Qubit fluorometer. Libraries were PCR amplified to incorporate index tags and flow cell-binding regions. Final libraries were quantified by Qubit, Bioanalzyer, and quantitative PCR (qPCR) and then sequenced via a 300-bp paired-end run on a MiSeq instrument using V3 chemistry and standard Illumina analysis software.

### Bioinformatic analysis.

For draft genomes, MiSeq reads were screened and trimmed based on length and quality using BBDUK within the Geneious software package, version R9 (Biomatters, Ltd.). The trimming process also removed any residual adapter sequences. Trimmed paired reads (4 × 10^5^ to 1.2 × 10^6^) were assembled *de novo* into contigs with the Geneious assembler. The number of reads used in each assembly was sufficient to give a minimum of 25-fold coverage, averaged across all contigs. (A maximum of 45-fold coverage was used.) Contigs that were <1 kb in length, with <2-fold read coverage, or with <80% high-quality base calls were eliminated from subsequent analysis. Annotation of contigs for the analysis presented here was done by the RAST server (34), but sequences submitted to GenBank were annotated by the NCBI prokaryotic genome annotation pipeline. Previously assembled genomes of ST95 ExPEC isolates from the Seattle area (5) were downloaded from NCBI for analysis. Prediction of acquired antibiotic resistance genes employed ResFinder v.2.1 (20). Identification of potential plasmid replicons was done with PlasmidFinder v.1.3 (28). Identification of insertion elements was done with ISFinder (35), and identification of lysogenic prophage used PHAST (36).

To determine whether a pUTI89-like plasmid was present in ST95 isolates from Seattle (5), pUTI89 was used to query the archived draft genomes using BLAST. Contigs or fragments greater than 500 bp (allowing for the possibility of misassembly) that were >98% identical to the pUTI89 sequence were aggregated. If >98% of the total 114-kb pUTI89 sequence could be generated by combining nonoverlapping contigs or fragments from an isolate, we considered that isolate to be positive for the pUTI89 plasmid.

Plasmids less than 10 kb were not well represented in the long-read data, as the template DNA had been size selected to be greater than 5 kb. These plasmids emerged from *de novo* assembly of 300-base MiSeq reads. Contigs were designated “small plasmids,” analogous to the “small cryptic plasmids” identified in ESBL-producing *E. coli* strains by Brolund et al. (37), if (i) they assembled *de novo* as circular sequences, (ii) they showed read coverage at least 50% higher than the mean coverage of the top five chromosomal contigs in the same assembly, (iii) they were <10 kb in size, and (iv) BLAST searches of GenBank identified primarily known plasmids, and the contig used as the query covered 80% or more of the plasmid sequence in GenBank. (The Geneious *de novo* assembler can produce a circular contig if the reads on each end of a linearly assembled contig match and those reads do not intersect with each other anywhere else in the contig.) The archived genomes of the Seattle isolates could not be used for identification of small plasmids through this approach, as topology and read coverage data were not available for these assemblies.

### Statistical analysis.

Comparison of population frequencies (e.g., frequency of pansusceptibility) and calculated *P* values used the chi-square test, with a significance cutoff of *P* = 0.05. Comparison of population means (e.g., mean number of small plasmids per isolate) used Students *t* test, with a significance cutoff of *P* = 0.05.

### Plasmid DNA isolation and transformation.

Plasmid DNA was isolated from *E. coli* cultures using the ZR Plasmid Miniprep Classic kit (Zymo Research). Plasmid DNA was analyzed on 1% agarose gels. DNA was transformed in chemically competent OneShot *E. coli* TOP10 cells (Invitrogen) by the manufacturer’s protocol, and colonies were selected on LB agar plus ampicillin (50 μg/ml).

### Accession number(s).

Complete genome sequences have been deposited in DDBJ/EMBL/GenBank under the following accession numbers: SF-468 chromosome and plasmids, CP012625 to CP012630; SF-173 chromosome and plasmid, CP012631 and CP012632; SF-166 chromosome and plasmid, CP012633 and CP012634; and SF-088 chromosome and plasmids, CP012635 to CP012638. The whole-genome shotgun sequencing projects described here have been deposited in DDBJ/ENA/GenBank under the accession numbers shown in Table S3.

10.1128/mSphere.00390-16.5 TABLE S3  GenBank accession numbers for *Escherichia coli* ST95 draft genome sequences reported in this work. Download TABLE S3, DOCX file, 0.1 MB. Copyright © 2017 Stephens et al. 2017 Stephens et al. This content is distributed under the terms of the Creative Commons Attribution 4.0 International license.
