# Supplementary material for: Genomic Analysis of Factors Associated with Low Prevalence of Antibiotic Resistance in Extraintestinal Pathogenic Escherichia coli Sequence Type 95 Strains
Source: mSphere. 2017 Apr 5;2(2):e00390-16. doi: 10.1128/mSphere.00390-16 (PMC5381267; doi:10.1128/mSphere.00390-16)
Supplement: TABLE S1 [file sph002172260st1.docx]

**Supplementary Table S1.** Overview of *E. coli* ST95 genomes sequenced for this work ^a^.

|  | ***fimH-1 strains*** | | ***fimH-6 strains*** | | ***fimH-9 strains*** | | ***fimH-47 strains*** | |
| --- | --- | --- | --- | --- | --- | --- | --- | --- |
|  | **SF-468 ^b^** | **Drafts**  **(n = 17)** | **SF-166 ^b^** | **Drafts**  **(n = 25)** | **SF-088 ^b^** | **Drafts**  **(n = 2)** | **SF-173 ^b^** | **Drafts**  **(n = 5)** |
| Total genome size (bp) | 5,142,799 bp chromosome,  215,844 bp plasmids (total) | 5.21 Mb | 4,910,962 bp chromosome, 114,331 bp plasmid | 5.16 Mb | 5,048,243 bp chromosome, 158,754 bp plasmids (total) | 5.11 Mb | 5,059,732 bp chromosome, 93,074 bp plasmid | 5.23 Mb |
| Mean number of contigs > 1 kb in assemblies | 1 chromosome, 5 plasmids | 50 | 1 chromosome, 1 plasmid | 34 | 1 chromosome, 3 plasmids | 38 | 1 chromosome, 1 plasmid | 45 |
| Mean contig length | - | 109 kb | - | 164 kb | - | 136 kb | - | 121 kb |
| Maximum contig length | 5,142,799 bp chromosome | 645 kb | 4,910,962 bp chromosome | 807 kb | 5,048,243 bp chromosome | 836 kb | 5,059,732 bp chromosome | 652 kb |
| Mean N_50_ | - | 288 kb | - | 407 kb | - | 244 kb | - | 330 kb |
| Mean predicted antibiotic resistance genes (range) | 8 | 3.1 (0-12) | 0 | 0.9 (0-8) | 5 | 2.5 (0-5) | 5 | 3.7 (1-5) |

^a^ Assembled genomes from Seattle ST95 isolates (5) are not included.

^b^ Genome fully assembled from SMRT sequencing data.
