# Supplementary figures and images for: Genomic Analysis of Factors Associated with Low Prevalence of Antibiotic Resistance in Extraintestinal Pathogenic Escherichia coli Sequence Type 95 Strains
Source: mSphere. 2017 Apr 5;2(2):e00390-16. doi: 10.1128/mSphere.00390-16 (PMC5381267; doi:10.1128/mSphere.00390-16)

**
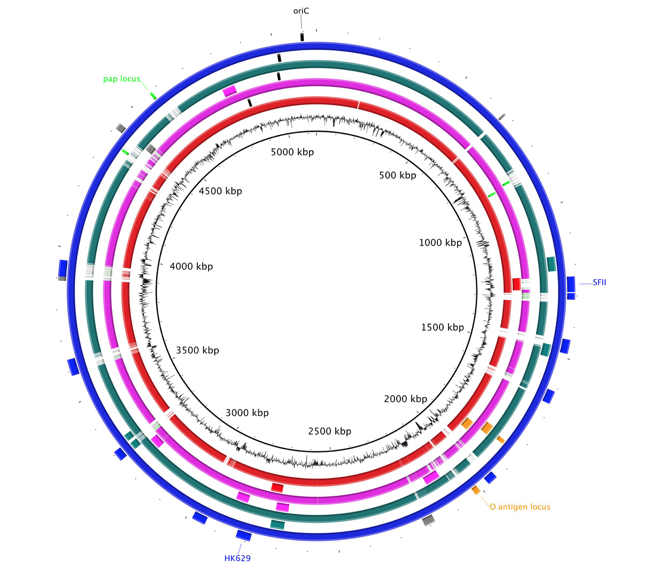
**

Supplement: FIG S1 [file sph002172260sf4.docx]

**Supplementary Figure S2**

**
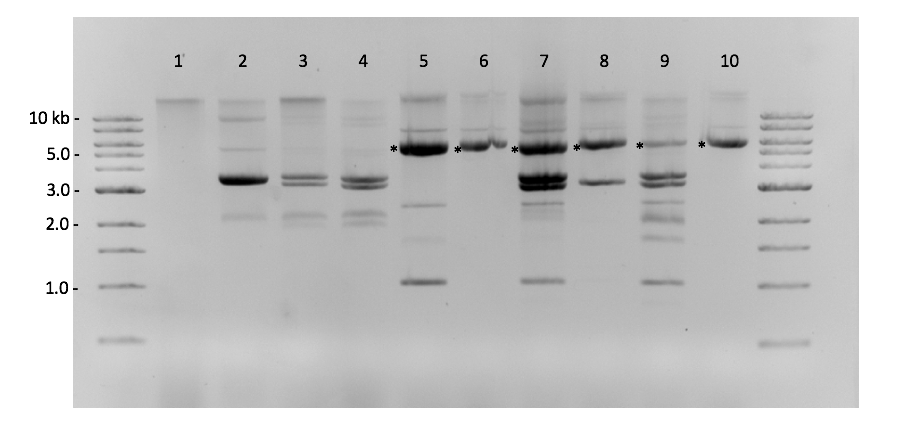
**

Supplement: FIG S2 [file sph002172260sf5.docx]
