# Supplementary material for: Genomic Analysis of Factors Associated with Low Prevalence of Antibiotic Resistance in Extraintestinal Pathogenic Escherichia coli Sequence Type 95 Strains
Source: mSphere. 2017 Apr 5;2(2):e00390-16. doi: 10.1128/mSphere.00390-16 (PMC5381267; doi:10.1128/mSphere.00390-16)
Supplement: TABLE S2 [file sph002172260st2.docx]

**Supplementary Table S2.** DNA methylation motifs identified in ST95 isolates through SMRT sequencing.

| **Motif** | **Methylated base** | **Putative MTase** | **Frequency of methylation** | | | |
| --- | --- | --- | --- | --- | --- | --- |
|  |  |  | **SF-468 (*fimH*-1)** | **SF-173 (*fimH*-47)** | **SF-088**  **(*fimH*-9)** | **SF-166 (*fimH*-6)** |
| GATC^a^ | m6A | Dam | + (100%) | + (100%) | + (100%) | + (100%) |
| GAAGNNNNNNNTGG^a^ | m6A |  | + (100%) | + (100%) | - | - |
| GAGACC | m6A |  | - | - | + (100%) | + (100%) |
| CAAACC | m6A |  | - | - | + (100%) | - |
| CACNNNGTA | m6A |  | - | - | + (100%) | - |
| RCCGGY^a^ | m4C |  | - | - | - | + (12%) |

^a^ Methylated bases detected on both strands of dyad symmetric motif.
