# Supplementary material for: Genomic Analysis of Factors Associated with Low Prevalence of Antibiotic Resistance in Extraintestinal Pathogenic Escherichia coli Sequence Type 95 Strains
Source: mSphere. 2017 Apr 5;2(2):e00390-16. doi: 10.1128/mSphere.00390-16 (PMC5381267; doi:10.1128/mSphere.00390-16)
Supplement: TABLE S3 [file sph002172260st3.docx]

**Supplementary Table S3.** GenBank accession numbers for *Escherichia coli* ST95 draft genome sequences reported in this work.

| ***E. coli* Isolate** | **Accession #** |
| --- | --- |
| SF-001 | MDFO00000000 |
| SF-075 | MDFQ00000000 |
| SF-083 | MDFP00000000 |
| SF-094 | MDGC00000000 |
| SF-095 | MKJI00000000 |
| SF-126 | MKJJ00000000 |
| SF-149 | MDFR00000000 |
| SF-151 | MDFS00000000 |
| SF-194 | MDSF00000000 |
| SF-231 | MDFT00000000 |
| SF-239 | MDFU00000000 |
| SF-264 | MDFV00000000 |
| SF-269 | MDFW00000000 |
| SF-305 | MDFX00000000 |
| SF-313 | MDFY00000000 |
| SF-335 | MDFZ00000000 |
| SF-356 | MDGA00000000 |
| SF-362 | MDGB00000000 |
| SF-371 | MDGD00000000 |
| SF-380 | MDGE00000000 |
| SF-383 | MDGF00000000 |
| SF-384 | MDXB00000000 |
| SF-403 | MDGG00000000 |
| SF-421 | MDGH00000000 |
| SF-423 | MDGJ00000000 |
| SF-425 | MDGI00000000 |
| SF-440 | MDSG00000000 |
| SF-452 | MDXC00000000 |
| SF-457 | MDSE00000000 |
| SF-491 | MKIX00000000 |
| SF-495 | MKIY00000000 |
| SF-501 | MKIZ00000000 |
| SF-518 | MKJA00000000 |
| SF-522 | MKJB00000000 |
| SF-522 | MKJB00000000 |
| SF-523 | MKJC00000000 |
| SF-560 | MKJD00000000 |
| SF-567 | MKJE00000000 |
| SF-572 | MKJF00000000 |
| SF-596 | MKJG00000000 |
| SF-626 | MKJH00000000 |
| MVAST0098 | MKJK00000000 |
| MVAST0176 | MKJL00000000 |
| MVAST0234 | MKJM00000000 |
| MVAST0326 | MKJN00000000 |
| USVAST184 | MKJO00000000 |
| USVAST245 | MKJP00000000 |
| USVAST267 | MKJQ00000000 |
| USVAST356 | MKJR00000000 |
| USVAST406 | MKJS00000000 |
